# Beyond the Blood: CSF-Derived cfDNA for Diagnosis and Characterization of CNS Tumors

**DOI:** 10.3389/fcell.2020.00045

**Published:** 2020-02-18

**Authors:** Abbye E. McEwen, Sarah E. S. Leary, Christina M. Lockwood

**Affiliations:** ^1^Department of Pathology, University of Washington, Seattle, WA, United States; ^2^Department of Laboratory Medicine, University of Washington, Seattle, WA, United States; ^3^Brotman Baty Institute for Precision Medicine, Seattle, WA, United States; ^4^Seattle Children’s Hospital, Cancer and Blood Disorders Center, Seattle, WA, United States; ^5^Department of Pediatrics, University of Washington, Seattle, WA, United States; ^6^Fred Hutchinson Cancer Research Center, Seattle, WA, United States

**Keywords:** cell-free DNA, cfDNA, ctDNA, liquid biopsy, brain tumor, biomarker, cerebrospinal fluid, CSF

## Abstract

Genetic data are rapidly becoming part of tumor classification and are integral to prognosis and predicting response to therapy. Current molecular tumor profiling relies heavily on tissue resection or biopsy. Tissue profiling has several disadvantages in tumors of the central nervous system, including the challenge associated with invasive biopsy, the heterogeneous nature of many malignancies where a small biopsy can underrepresent the mutational profile, and the frequent lack of obtaining a repeat biopsy, which limits routine monitoring to assess therapy response and/or tumor evolution. Circulating tumor, cell-free DNA (cfDNA), has been proposed as a liquid biopsy to address some limitations of tissue-based genetics. In cancer patients, a portion of cfDNA is tumor-derived and may contain somatic genetic alterations. In central nervous system (CNS) neoplasia, plasma tumor-derived cfDNA is very low or absent, likely due to the blood brain barrier. Interrogating cfDNA in cerebrospinal fluid (CSF) has several advantages. Compared to blood, CSF is paucicellular and therefore predominantly lacks non-tumor cfDNA; however, patients with CNS-limited tumors have significantly enriched tumor-derived cfDNA in CSF. In patients with metastatic CNS disease, mutations in CSF cfDNA are most concordant with the intracranial process. CSF cfDNA can also occasionally uncover additional genetic alterations absent in concurrent biopsy specimens, reflecting tumor heterogeneity. Although CSF is enriched for tumor-derived cfDNA, absolute quantities are low. Highly sensitive, targeted methods including next-generation sequencing and digital PCR are required to detect mutations in CSF cfDNA. Additional technical and bioinformatic approaches also facilitate enhanced ability to detect tumor mutations in CSF cfDNA.

## Introduction

The incidence of primary brain tumors in the United States is approximately 30 per 100,000 persons; one third of these tumors are malignant. The overall 5-year survival for malignant primary brain tumors is 30% and can be as low as ∼5% in the most common aggressive subtype, glioblastoma (GBM) (Ostrom et al., 2018). In children, primary brain and spinal cord tumors are the most common childhood solid tumor malignancy and the most common cause of cancer related death in children under 15 years (Siegel et al., 2017; Withrow et al., 2018). The diagnosis of primary brain tumors has become increasingly dependent upon molecular tumor profiling, with a number of tumors now defined by key molecular alterations (Louis et al., 2016). Many molecular biomarkers are already being used in clinical practice for prognosis and to guide targeted therapy (Szopa et al., 2017). This number will only increase with the development of more targeted therapies. Tumors that have metastasized to the brain are often resistant to treatment and have a low 5-year survival rate (2.4% across all tumor types) (Hall et al., 2000; Brastianos et al., 2015; Paik et al., 2015; Preusser et al., 2018).

Current techniques for molecular profiling of brain tumors primarily rely on tissue obtained through small biopsies or surgical resection. Brain biopsy is an invasive procedure, which carries a risk of mortality reported to be between 2.8 and 12% depending on technique and patient population (Yong and Lonser, 2013; Malone et al., 2015). Moreover, tumors in certain anatomic locations such as the brain stem are dangerous to biopsy or resect (Kickingereder et al., 2013; Malone et al., 2015).

Liquid biopsy techniques are fast emerging as non-invasive methods for tumor diagnosis and molecular characterization. Cell-free DNA (cfDNA) refers to DNA that has entered the circulation after cell death. In healthy patients, the majority of this DNA comes from the apoptosis of hematopoietic cells and has a length of ∼167 base pairs. The total amount of circulating cfDNA is increased in patients with solid tumors (Leon et al., 1977), and the tumor-derived cfDNA is also shorter in length (∼145 bp) (Mouliere et al., 2011; Zheng et al., 2012). cfDNA is also present in other body fluids such as sputum, stool, saliva, urine, pleural fluid, and cerebrospinal fluid (CSF) (Peng et al., 2015). CSF is an appealing source for liquid biopsy of CNS tumors as it may be obtained through the minimally invasive procedure of diagnostic lumbar puncture, which is performed routinely for many neurologic diseases with a very low serious complication rate (Doherty and Forbes, 2014). Diagnostic lumbar puncture is clinically indicated for CNS tumor staging to evaluate CSF cytology in some tumors such as medulloblastoma with a known rate of metastatic CSF dissemination (Juraschka and Taylor, 2019). This review will discuss the use of cell-free DNA in brain and spinal cord tumor diagnosis and characterization and the advantages of using CSF as the sample of choice. Preanalytical variables and detection strategies are also covered.

## cfDNA From CSF Is More Sensitive Than Plasma in Diagnosing Brain Neoplasia

Circulating tumor-derived cell-free DNA has emerged as a non-invasive modality for genetic characterization of many solid tumors. Advantages of using cell free DNA include that it is present in the plasma of most patients and that it allows serial monitoring of tumor burden and response to therapy. In a study of 640 patients, tumor-derived cfDNA was detected in the blood of >75% of patients with many types of advanced carcinomas (stage III/IV as indicated in the paper) and in approximately 50–75% of patients with localized carcinomas. In contrast, it was detected in only 40% of medulloblastoma patients and in <10% of patients with gliomas. Moreover, the absolute concentration of tumor-derived cfDNA in glioma and medulloblastoma patients was several logs lower than in patients with other tumor types (Bettegowda et al., 2014). cfDNA detection has been reported to be more likely in the plasma of patients with large and/or contrast enhancing tumors (Boisselier et al., 2012). A subsequent study of 35 patients, all with primary CNS neoplasia, found that tumor-derived cfDNA could be detected in the CSF of nearly 75% of patients, including all high-grade tumors that were in direct contact with the CSF space (Wang et al., 2015). Another study of 10 patients (7 with solid brain tumors and 3 with leptomeningeal metastases) reported that the median concentration of total cfDNA was lower in CSF than in plasma. However, in the absence of widely disseminated disease, mutant allele frequencies were significantly higher in the CSF (Pan et al., 2015). This observation was also reported in an additional study of 12 patients (4 with glioblastoma, 6 with metastatic breast carcinoma, and 2 with metastatic lung carcinoma) (De Mattos-Arruda et al., 2015).

## Csf-Derived cfDNA Offers Additional Genetic Information Beyond Tissue Biopsy

Genetic analysis of small biopsy specimens may not reflect intratumor heterogeneity or uncover mutations only present at a specific metastatic site (Gerlinger et al., 2012). Intratumoral heterogeneity is defined by the presence of different mutations in different parts of the same tumor and is a well-known feature of high-grade glioma (Sottoriva et al., 2013; Aubry et al., 2015; Mahlokozera et al., 2017) and medulloblastoma (Morrissy et al., 2017). A study of 10 patients with newly diagnosed glioblastoma whose tumors were sequenced in different sectors demonstrated that 51% of mutations were clonal (present in all tumor cells), 3% were subclonal (present in a subset of tumor cells) but shared between different sectors, and that 46% of mutations were subclonal and unique to a particular sector. Moreover, 80% of patients had potentially targetable mutations that were not shared between sectors, which highlights the danger of relying on single-sector sequencing information (Mahlokozera et al., 2017).

Using CSF-derived cell free DNA may overcome the under-sampling bias that may occur with small tissue biopsies. In patients with CNS restricted disease (primary or metastatic), mutations have been found in CSF or plasma cfDNA that are not present in the corresponding biopsy specimen (De Mattos-Arruda et al., 2015; Pan et al., 2015; Mouliere et al., 2018). In the context of disseminated disease, mutations in CSF cfDNA recapitulate brain and meningeal metastasis that are not found in plasma cfDNA or extracranial metastases (De Mattos-Arruda et al., 2015).

**TABLE 1:** Comparison of technology platforms for detection of cell-free DNA.

***Method***	***Analytical sensitivity***	***Mutations***	***Targets per assay***	***Mutation info required?***	***Turnaround time***	***Cost***
**PCR-Based**						
Digital PCR^a^	0.01–0.1%	SNV, indels, CNV, structural	1–4	Yes	Fast	$
BEAMing^b^	0.01%	SNVs, indels	1–20	Yes	Fast	$
**Next generation sequencing**						
**Targeted panels**						
Deep sequencing^c^	0.05–0.1%	SNV, indels	Panel	No	Intermediate	$$$
Error corrected NGS^d^	<0.02%	SNV, indels	Panel	No	Intermediate	$$$
TAm-Seq^e^	<0.1%	SNV, indels	Panel	No	Intermediate	$$$
CAPP-Seq^f^	<0.02%	SNV, indels, structural, CNV	Panel	No	Intermediate	$$$
cSMART^g^	0.01%	SNV, indels, structural	Panel	No	Intermediate	$$$
**Whole genome/exome**						
Traditional^h^	5 – 10%	SNV, indels, structural variants, CNV	Genome	No	Intermediate (analysis is complex)	$$$$
Low-Pass^i^		Structural variants, CNV	Genome	No	Fast	$$

*Platforms are divided by the scale of analysis performed. References: ^*a*^Watanabe et al., 2015; ^*b*^Dressman et al., 2003; ^*c*^Miller et al., 2019; ^*d*^Narayan et al., 2012; ^*e*^Forshew et al., 2012; ^*f*^Newman et al., 2014; ^*g*^Wang et al., 2017; ^*h*^Adalsteinsson et al., 2017; ^*i*^Ge et al., 2019.*

## Clinical Applications

### Glioma Classification

Gliomas are the most common primary brain tumor and are clinically characterized by several key molecular markers in addition to histology and grade. The histologic diagnoses within glioma, including astrocytoma, glioblastoma, and oligodendroglioma, are each further characterized by *IDH* mutation status. Oligodendroglioma is identified by 1p/19q co-deletion, and both astrocytoma and oligodendroglioma are further described as histologic grade II or III, while glioblastoma is by definition grade IV (Louis et al., 2016). Methylation status of *MGMT* is another key prognostic marker in glioblastoma, with methylated tumors conferring sensitivity to therapy with temozolomide (Hegi et al., 2005). Genomic analyses have demonstrated multiple abnormalities such as activating kinase mutations in *EGFR* and *PDGFRA* in malignant glioma, but the success of targeted therapy has been limited to date, which may be at least in part due to tumor heterogeneity (Paolillo et al., 2018). Diffuse midline glioma, H3K27M-mutant is a newly described entity that occurs mainly in the pediatric age range (Sturm et al., 2012). This tumor is defined by the pathognomonic H3K27M mutation in the *H3F3A* or, less commonly, *HIST1H3B* genes, and all glial tumors with H3K27M are considered grade IV regardless of histologic appearance. In the pediatric age range, low-grade gliomas are more common than high-grade. Pilocytic astrocytoma, a grade I tumor commonly harboring abnormalities of *BRAF*, *FGFR*, or *NF1*, is the most common pediatric brain tumor, and new targeted therapies are expanding therapeutic options (Olow et al., 2016). An integrated histologic and molecular diagnosis is strongly prognostic in glioma, with survival ranges between 1 and 15 years (Louis et al., 2016). While surgery continues to be an essential component of initial diagnosis and treatment when feasible, monitoring for tumor recurrence is currently by MRI, which is not sensitive for microscopic disease (Aquino et al., 2017). A panel of seven genes (*IDH1*, *IDH2*, *TP53*, *TERT*, *ATRX*, *H3F3A*, and *HIST1H3B*) has been developed that can accurately classify nearly 80% of malignant gliomas based on genetics alone. A retrospective study of 20 glioma patients demonstrated that this combined digital PCR and targeted sequencing panel could use CSF cfDNA to correctly classify gliomas in 85% of cases and only failed with low grade tumors or those not in direct contact with the ventricles (Martínez-Ricarte et al., 2018).

### Brainstem Tumors

Many tumors of the brain stem have characteristic alterations (Dunham, 2015; Kristensen et al., 2019). Perhaps the most successful use for CSF cfDNA has been in the characterization of brain stem tumors, which are inherently difficult and dangerous to biopsy. In a study of 57 patients, a next-generation sequencing (NGS) panel of 68 genes commonly mutated in brain stem tumors detected tumor specific mutations in the CSF cfDNA of 82.5% of patients. Consistent with molecular findings from tumor-based studies, two markers in this panel were shown to correlate with overall survival (OS) in patients with diffuse midline glioma in this cohort — the presence of an *H3F3A/HIST1H3B* mutation was predictive of poor overall survival consistent with the specific entity of H3K27M-mutant diffuse midline glioma, where as an *IDH1* mutation predicted better OS (Pan et al., 2019). Targeted therapies for these alterations are currently undergoing clinical trials (Long et al., 2017). *Histone 3* allele-specific PCR and single gene Sanger sequencing assays have been developed to aid the diagnosis of H3K27M-mutant diffuse midline gliomas, with 87.5% clinical sensitivity for CSF cfDNA when compared to tissue testing (Chen et al., 2015).

### Monitoring Response to Therapy

Brain tumor patients are usually biopsied once during the course of their illness. In patients with primary brain tumors or CNS metastases, mutant allele frequencies have been shown to decrease with surgical and systemic therapy and correlate with tumor burden (De Mattos-Arruda et al., 2015). Therefore, there is potential that CSF cfDNA could serve as a useful biomarker for monitoring tumor progression and response to therapy (Miller et al., 2019). Moreover, genomic alterations that drive the growth of glioma recurrence are distinct from those present in the initial resection, likely due to tumor evolution (Johnson et al., 2014). Monitoring patients with CSF cfDNA may therefore provide opportunity to provide targeted therapy to tumor recurrences.

cfDNA may prove especially useful in monitoring high-grade gliomas, which present a unique diagnostic challenge. Pseudoprogression of brain tumors is defined by radiographic alterations that are due to treatment rather than tumor growth, including increased lesion size, contrast enhancement, and/or edema, in the absence of increased tumor activity. With the advent of immunotherapy, disease monitoring by imaging alone has become increasingly complicated as the tempo and MRI appearance of tumor progression and response can be less predictable with the use of immunotherapy than traditional cytotoxic therapy (Aquino et al., 2017). Although pseudoprogression often resolves on its own, in some cases it progresses to treatment-related necrosis (Brandsma and van den Bent, 2009; Wen et al., 2017). Definitive diagnosis requires a tissue biopsy, which may be difficult to interpret in the setting of extensive necrosis and therapy-related cytologic atypia (Perry and Schmidt, 2006).

### CNS Lymphoma

Primary CNS lymphoma (PCNSL) is part of the differential diagnosis for many space occupying brain lesions (Smirniotopoulos and Goldstein, 2012), and in contrast to solid primary CNS solid tumors, surgical management is not routinely part of treatment (von Baumgarten et al., 2018). In a small study of patients with PCNSL, *MYD88* L265P mutations were detected by droplet digital PCR in plasma cfDNA in 8 of 14 patients known to harbor this mutation in their tumors (Hattori et al., 2017). A similar study of PCNSL patients using a targeted NGS panel detected patient specific mutations in the plasma cfDNA in only 32% of patients, including *MYD88* mutations in 8 of 20 cases (Fontanilles et al., 2017). CSF cytology and immunophenotyping by flow cytometry are currently used as alternatives to stereotactic biopsy in patients with suspected PCNSL. Using CSF as a source for cfDNA shows increased clinical sensitivity relative to plasma cfDNA testing (86%), and *MYD88* L265P mutations may be detected even in the absence of positive cytology or flow cytometry (Rimelen et al., 2019). *MYD88* L265P mutations are specific to PCNSL and have not been reported in other brain tumors such as GBM (Nakamura et al., 2016; Fontanilles et al., 2017), making this a particularly appealing biomarker. In the future, it may be possible to render the diagnosis of PCNSL based on a *MYD88* L265P molecular result even in the absence of positive cytology.

### Brain and Leptomeningeal Metastasis

Brain and leptomeningeal metastases are common in certain cancers and carry a very poor prognosis. They are difficult to biopsy and often resistant to treatment. In a study of lung adenocarcinoma patients with leptomeningeal metastasis and known *EGFR* mutations in the primary tumor, patient-specific EGFR mutations were detected in the CSF cfDNA of all 26 patients. Using CSF cfDNA had greater clinical sensitivity than plasma cfDNA or DNA derived from direct cellular lysis of the CSF (86.4 and 73.1%, respectively). Furthermore, the average mutant allele frequencies were highest in CSF-derived cfDNA (62%, vs. 13.9% for direct lysis and 3.5% for plasma), making it more likely that mutations were accurately detected. Many copy number variants were detectable only in the CSF cfDNA and were more abundant in patients who failed tyrosine kinase inhibitor therapy. Common molecular copy number alterations were present in *MET*, *KRAS*, *ERBB2*, *BRAF*, and *ALK*. *EGFR* T790M was identified in the CSF of 30.4% of patients with progression on tyrosine kinase inhibitor treatment vs. 21.7% in plasma (Li et al., 2018). An additional study of 29 lung cancer patients with leptomeningeal metastases revealed CNS-limited single nucleotide variants (SNVs) in *ALK* and *KRAS* (Ge et al., 2019). Similar CNS-limited drug resistance mutations have been described in the setting of melanoma and breast carcinoma (Pentsova et al., 2016).

Current definitive diagnosis of leptomeningeal metastasis depends upon CSF cytology, which can be falsely negative in up to 20% of cases with positive clinical and radiographic findings (Glantz et al., 1998; Clarke et al., 2010). Tumor-derived cfDNA has been reported to be present in the CSF of breast (De Mattos-Arruda, 2017) and lung carcinoma patients (Swinkels et al., 2000) even in the absence of cytologically malignant cells. One group has shown that the Cobas^®^ (Roche) EGFR Mutation Test v2, an approved companion diagnostic for osimertinib, can detect EGFR mutations, including T790M, from the supernatant cfDNA from CSF cytology samples with 87.5% clinical sensitivity and 100% specificity (Kawahara et al., 2019).

### Preanalytical Variables

All laboratory testing consists of pre-analytic, analytic, and post-analytic phases. Pre-analytical variables refer to those that occur prior to testing and include physiologic factors intrinsic to the patient and factors that affect specimen integrity such collection, processing, and storage (Dale, 1998; Narayanan, 2000). Physiologic factors include those that limit the amount of tumor-derived cfDNA present in the CSF. Multiple studies have shown that low grade tumors or those not directly communicating with the CSF space have low to undetectable tumor-derived cfDNA in the CSF (Wang et al., 2015; Juratli et al., 2018; Martínez-Ricarte et al., 2018; Pan et al., 2019). Likewise, detection of tumor-derived cfDNA is less likely in patients with new onset disease (Pan et al., 2019). Studies of CSF cytology have shown that obtaining CSF as close to the tumor as possible minimizes the risks of false negative results (Chamberlain et al., 2001), though the effect on cfDNA yield has yet to be determined.

Analytical success is dependent upon maintaining cfDNA integrity and minimizing contamination from cellular genomic DNA. However, rigorous studies of these factors in CSF are lacking. In contrast, pre-analytic variables that affect the detection of plasma cfDNA have been investigated. At present, the best practices for handling CSF cfDNA must be inferred from studies of plasma. In standard K_2_EDTA blood collection tubes, it has been shown that separating plasma from cellular components (through centrifugation or filtration) should be performed as soon as possible after collection, ideally within 6 h, to prevent dilution with leukocyte DNA (Merker et al., 2018). While we recommend expedient processing of CSF, this factor may not be as important in CSF as this fluid is intrinsically paucicellular. Special cell-stabilizing blood collection tubes have been used to minimize genomic DNA contamination, which allows processing to be delayed for multiple days (Norton et al., 2013; Diaz et al., 2016; Merker et al., 2018). Although some labs are using these for CSF (Hickmann et al., 2019), the practice is not standard, and the value of cell-stabilizing tubes has not been systematically investigated. As with plasma, CSF specimens can be stored frozen after processing, but multiple freeze-thaw cycles should be avoided (Merker et al., 2018).

Multiple methods for purifying cell free DNA exist, but none of these have been systematically studied in CSF. For example, Hickmann et al. (2019) reported that in their general experience, the QIAamp Circulating Nucleic Acid Kit provided better results with CSF. Likewise, Huang et al. (2017) reported that centrifugation for 10 min at 1000 × *g* is optimal for selection of DNA fragments of ∼150 bp, but others have not confirmed this.

### Analytical Strategies: Targeted vs. Broad Approaches

Methods for analyzing cfDNA can be divided into two broad categories — targeted approaches for interrogating a single or few variants and broad approaches that cover a panel of genes up to an entire genome. Targeted assays typically use PCR-based techniques, or in rare cases, single gene sequencing. These assays are inexpensive, have a fast turn-around time, and are easy to interpret. Multiple qPCR and modified PCR assays are available, including BEAMing (Dressman et al., 2003) and the companion diagnostics Therascreen (Qiagen) and Cobas (Roche) (Reck et al., 2016), which have an analytical sensitivity of 0.01 to 0.1%. Digital PCR methods allow for quantification of tumor-derived cfDNA levels and can be used to detect copy number variations (Mazaika and Homsy, 2014; Watanabe et al., 2015). These methods usually require prior knowledge of patient specific mutations present in previous biopsies and can be used to monitor disease progression or response to treatment. They can also be used to answer specific directed questions. For example, this approach can be used when knowing the presence or absence of a single genetic change will provide a diagnosis (see the discussion above of PCNSL and brainstem tumors), direct targeted therapy, i.e., *MEK* inhibitors in *BRAF* V600E mutated CNS melanoma (Melms et al., 2018), or provide prognostic information, i.e., *TERT* promoter variants in malignant gliomas (Shankar et al., 2015). Multiple PCR assays can be combined into small panels with relatively fast turn-around time, but analytical sensitivity may be impacted (see discussion above on glioma classification).

Early studies of the analytic validity of CSF-derived cfDNA relied on these targeted methods and compared the presence of known patient specific mutations in paired tissue biopsies and CSF specimens. In clinical practice, this approach is not always feasible, particularly in cases where CSF-derived cfDNA is needed for primary diagnosis. Moreover, targeted approaches will not provide information about tumor heterogeneity or CNS-specific mutations. Targeted massively parallel NGS approaches use PCR amplification or hybrid capture enrichment to focus on a particular subset of the genome and do not rely upon *a priori* knowledge of tumor alterations. However, these assays are more complicated, expensive, and increase turn-around time. In general, analytical sensitivity decreases as the number of targets increases and is optimal with targets totaling less than 200 kilobases (Newman et al., 2016). The most sensitive NGS assays (<0.02%) employ molecular barcoding and error correction and surpass the most sensitive PCR-based assays (Narayan et al., 2012; Newman et al., 2016). This degree of analytical sensitivity may not be universally required for CSF cfDNA; however, molecular barcoding is beneficial in the context of limited DNA input, commonly encountered in the CSF, and in clinical settings of coincident inflammation and/or infection.

Both general cancer gene panels and brain tumor-specific panels have been used to analyze CSF cfDNA. Using an NGS panel of all exons in 341 cancer associated genes with actionable mutations (MSK-IMPACT), alterations were detected in 63% of patients with CNS metastases and 50% of patients with primary brain tumors (Pentsova et al., 2016). The published limit of detection for this panel is between 2–5% for low frequency variants (Cheng et al., 2015). In a more limited panel of all exons of 67 brain-tumor associated genes and the *TERT* promoter, alterations were detected in 82.5% of patients with brainstem tumors with an analytical sensitivity of 0.15% (Pan et al., 2019). A study looking at *EGFR* mutations in the CSF of lung cancer patients with CNS metastases showed that sequencing hotspots was more sensitive than sequencing all *EGFR* exons (Ge et al., 2019).

Non-targeted whole genome or exome sequencing has an analytical sensitivity of approximately 5% (Heitzer et al., 2013; Adalsteinsson et al., 2017). At first glance, this broad approach seems appealing in the context of tumors which are inaccessible to biopsy for which we do not have *a priori* knowledge of patient-specific genetic changes. However, in a small study of patients with brain tumors in dangerous anatomic locations, known mutations could not be found in two of four subjects, both of which had variant allele frequencies below 1% (Wang et al., 2015). While traditional whole genome sequencing is not feasible in the context of limited cfDNA input, low-pass or shallow whole genome sequencing (∼0.1× coverage) can be used to detect amplifications and deletions (Ge et al., 2019). In a study of 13 patients with GBM, shallow whole-genome sequencing was able to detect copy number variants (CNVs) in the CSF cfDNA of 38% of patients (Mouliere et al., 2018).

## Conclusion and Future Directions

Cell-free DNA has emerged as a powerful biomarker for the diagnosis and characterization of tumors of the central nervous system. Using CSF as a source has several advantages over plasma, including increased clinical sensitivity and the ability to detect CNS-limited mutations. Moreover, cfDNA from CSF is more likely to represent tumor heterogeneity than tissue biopsy, and if sampled over time, allows for the ability to monitor tumor progression and detect tumor evolution and drug resistance mutations.

Most current studies of CSF cfDNA are small and retrospective. A few case reports have demonstrated that CSF cfDNA has been used clinically to guide (Melms et al., 2018; Guo et al., 2019) and monitor response to therapy (Siravegna et al., 2017). Prospective trials, however, are lacking. Pre-analytical variables need to be systematically studied and optimized. Since absolute quantities of cell-free DNA in the CSF are low, NGS panels need to be strategically designed to be broad enough to cover common mutations while narrow enough to maintain adequate analytical sensitivity.

It has recently been shown that the variation of nucleosome positioning between tissue types can be exploited in cell-free DNA to predict tissue of origin. Notably, this observation extends to malignant tissue, which could be leveraged to facilitate determination of tumor origin from cell-free DNA (Snyder et al., 2016). This may prove to be a powerful tool in the CSF as it would allow clinicians to determine tumor type in the absence of sequence information and potentially enable disease monitoring. Whether cell-free DNA in the CSF provides a complementary alternative to tissue biopsies in the near future remains an area of active investigation. However, the significant advantages of this analyte for CNS tumors and the rapidly expanding genomic toolbox make this a promising area for additional studies.

## Author Contributions

All authors conceptualized the content and wrote the manuscript.

## Conflict of Interest

The authors declare that the research was conducted in the absence of any commercial or financial relationships that could be construed as a potential conflict of interest.
